# EFFICACY OF THE STROLL SAFE OUTDOOR FALLS PREVENTION PROGRAM

**DOI:** 10.1093/geroni/igac059.1103

**Published:** 2022-12-20

**Authors:** Tracy Chippendale, Steven Albert

**Affiliations:** New York University, New York, New York, United States; University of Pittsburgh, Pittsburgh, Pennsylvania, United States

## Abstract

Informed by the Ecological and Health Belief models, the manualized Stroll Safe program addresses the unmet need of a targeted outdoors falls prevention program. We examined the efficacy of the program in partnership with eight Naturally Occurring Retirement Community program sites in one U.S city (N=86). Participants age 60 and older with a history of falls or a fear of falling were recruited. Community sites were randomly assigned to the treatment or wait list control group. Results of independent samples t-tests reveal a statistically significant improvement in knowledge of outdoor fall risks and safe community mobility strategy use for the treatment group as compared to the wait list control group on the Outdoor Falls Questionnaire (OFQ) and Falls Behavioral Scale for the Older Person (FaB) (ps<.001, Cohen’s d=1.9 & 1.2). Improvements were retained at 6-week follow up. Implementation strategies are being developed to facilitate program adoption by other occupational therapists.

